# Augmenting Engagement in Decentralized Clinical Trials for Atrial Fibrillation: Development and Implementation of a Programmatic Architecture

**DOI:** 10.2196/66436

**Published:** 2025-05-12

**Authors:** Toluwa Daniel Omole, Andrew Mrkva, Danielle Ferry, Erin Shepherd, Jessica Caratelli, Noah Davis, Richmond Akatue, Timothy Bickmore, Michael K Paasche-Orlow, Jared W Magnani

**Affiliations:** 1 University of Pittsburgh School of Medicine, 3609 Forbes Avenue, Second floor, Pittsburgh, PA, United States; 2 Department of Medicine, University of Pittsburgh, Pittsburgh, PA, United States; 3 Khoury College of Computer Sciences, Northeastern University, Boston, MA, United States; 4 Department of Medicine, Tufts University School of Medicine, Boston, MA, United States; 5 Center for Research on Health Care, Department of Medicine, University of Pittsburgh, 3609 Forbes Avenue, Pittsburgh, PA, 15213, United States, 1 4123830611

**Keywords:** atrial fibrillation, rurality, diversity, mobile health intervention, mobile health, mhealth, chronic cardiovascular condition, cardiovascular, cardio, heart, vascular, medication, self-monitoring, digital health, programmatic architecture, effectiveness, smartphone-based, smartphone, telehealth, telemedicine, digital technology, application, digital literacy, clinical trial, cardiovascular trials

## Abstract

**Background:**

Atrial fibrillation (AF) is a chronic cardiovascular condition that requires long-term adherence to medications and self-monitoring. Clinical trials for AF have had limited diversity by sex, race and ethnicity, and rural residence, thereby compromising the integrity and generalizability of trial findings. Digital technology coupled with remote strategies has the potential to increase recruitment of individuals from underrepresented demographic and geographic populations, resulting in increased trial diversity, and improvement in the generalizability of interventions for complex diseases such as AF.

**Objective:**

This study aimed to summarize the architecture of a research program using remote methods to enhance geographic and demographic diversity in mobile health trials to improve medication adherence.

**Methods:**

We developed a programmatic architecture to conduct remote recruitment and assessments of individuals with AF in 2 complementary randomized clinical trials, funded by the National Institutes of Health, to test the effectiveness of a smartphone-based relational agent on adherence to oral anticoagulation. The study team engaged individuals with either rural or metropolitan residences receiving care for AF at health care settings who then provided consent, and underwent baseline assessments and randomization during a remotely conducted telephone visit. Participants were randomized to receive the relational agent intervention or control and subsequently received a study smartphone with installed apps by mail. Participants received a telephone-based training session on device and app usage accompanied by a booklet with pictures and instructions accessible for any level of health or digital literacy. The program included remote methods by mail and telephone to promote retention at 4-, 8-, and 12-month visits and incentivized return of the smartphone following study participation. The program demonstrated excellent participant engagement and retention throughout the duration of the clinical trials.

**Results:**

The trials enrolled 513 participants, surpassing recruitment goals for the rural (n=270; target n=264) and metropolitan (n=243; target n=240) studies. A total of 62% (319/513) were women; 31% (75/243) of participants in the metropolitan study were African American, Asian, American Indian or Alaskan native or other races or ethnicities, in contrast to 5% (12/270) in the rural study. Among all participants, 56% (286/513) had less than an associate’s degree and 44% (225/513) were characterized as having limited health literacy. Intervention recipients receiving the relational agent used the agent median of 95‐98 (IQR, 56‐109) days across both studies. Retention exceeded 89% (457/513) at 12 months with study phones used for median 3.3 (IQR, 1‐5) participants.

**Conclusions:**

We report here the development and implementation of a programmatic architecture for the remote conduct of clinical trials. Our program successfully enhanced trial diversity and composition while providing an innovative mobile health intervention for medication adherence in AF. Our methods provide a model for enhanced recruitment and engagement of diverse participants in cardiovascular trials.

## Introduction

Multiple demographic groups have had limited participation in clinical trials, despite relatively high rates of disease burden [1]. Women, racial and ethnic minorities, and people who reside in rural settings have historically been underrepresented in randomized clinical trials testing or evaluating interventions for cardiovascular diseases [2-6]. Causes of such underrepresentation are multifactorial and related to the individual or patient, investigator, and health care system factors [1,7,8]. Atrial fibrillation (AF) is a chronic cardiovascular condition that merits attention because of its high prevalence and the documented disparities in disease detection, treatment, and outcomes [9]. Global and United States prevalence of AF has increased with concomitant rise in clinical adversity, expenditures, and mortality associated with the condition [10-12]. Individuals with AF experience 4-fold higher rates of inpatient care and 5-fold higher days of hospitalization than those without [13]. The estimated health care costs for AF total US $6‐20 billion annually, which underscores the importance of trial representation of inclusion of populations that may have increased risks of clinical adversity [14].

Social factors are related to disparities in patient care and experience of AF [15]. Racial and ethnic disparities in AF management are evidenced by Black individuals being less likely to receive oral anticoagulation–a mainstay for thromboembolic stroke prevention in AF–than counterparts of White race [16]. Rural residents may experience structural barriers to care, and in turn lower quality care compared to individuals residing in metropolitan settings [15]. Furthermore, AF is a complicated condition with expectations that patients self-monitor for symptoms, adhere consistently to complex therapies like oral anticoagulants, and have awareness about the disease-related complications [17,18]. Clinical trials likewise have potential to assess the contributions of social and structural factors to patient experience and outcomes in a chronic condition such as AF.

Digital and mobile health interventions have multiple advantages for clinical trials to address the challenges described here. In many circumstances, digital technology can obviate geographic barriers and thereby encourage participation by eliminating travel as a geographic barrier. Such an approach may particularly benefit rural individuals who would otherwise be required to travel as well as metropolitan residents who also experience transportation costs and obstacles [19]. Coupling digital interventions with decentralized trial administration has clear potential to augment the geographic and social diversity of clinical trial participants, which can in turn enhance the generalizability of results and generate new biomedical knowledge [20].

Here we present the design and architecture of a remote mobile health program. We describe a program that uses a smartphone-based intervention to augment the self-management of AF. The intervention incorporates a relational agent [21]—an animated health educator that uses synthetic speech and conversational gestures, such as hand movements, gaze shifts, natural pauses, and emphatic facial expressions to simulate face-to-face counseling—in conjunction with a mobile heart rhythm sensor. We describe here the programmatic architecture to conduct remote trials and the resulting augmentation of geographic, ethnic, and racial diversity of participants. Rather than summarize the results of 2 contrasting trials, our objective in this manuscript is to demonstrate a successful strategy to increase the social and geographic diversity of participants in technology-based trials.

## Methods

### Summary of Recruitment

Our program implemented 2 complementary, parallel-arm, and randomized clinical trials with decentralized administration, summarized here and described in further detail elsewhere [18,22,23]. One trial (ClinicalTrials.gov ID NCT04076020), conducted in individuals with a rural Pennsylvania residence as determined using a definition of rural status as designated by the United States Census Bureau, aimed to recruit geographically remote individuals with AF. The second trial (ClinicalTrials.gov ID NCT04075994) focused on recruitment of individuals residing in metropolitan communities with a focus on economically depressed regions of Pittsburgh, Pennsylvania. Both trials prioritized recruitment from populations that have historically had limited representation in clinical trials for AF. Eligibility for participation in either trial included a diagnosis of AF, as confirmed by the electronic health record, and the prescription of oral anticoagulation for the purpose of thromboembolic stroke prevention in the setting of having AF. The rural and metropolitan trials aimed to recruit 264 and 240 participants, respectively, given differences in design and complementary study objectives.

The architecture of this program consisted of entirely remote recruitment, engagement, assessment, and retention. In effect, this process resulted in the absence of in-person contact between participants and study team members. Recruitment for both studies occurred using multiple approaches. Foremost, eligible individuals received an introductory letter cosigned by their physician provider, such as a physician or nurse practitioner to introduce the research study, accompanied by a brochure, contact information, and a stamped postcard to decline participation. Individuals who did not return the postcard within 2‐4 weeks received a telephone call as described by the letter. Participants also self-referred, having learned about the study from their physicians or material placed in clinic settings. Those interested in participating underwent telephone-based screening to verify appropriateness for the trial and review of the inclusion and exclusion criteria summarized in, Textbox 1. If eligible, potential participants scheduled a baseline interview and were then mailed the informed consent and materials for the baseline visit.

Textbox 1. **Inclusion and exclusion criteria.**

**Inclusion criteria**

- Adult, age≥18 years.
- Diagnosis of AF, identified from the electronic health record problem list with confirmation by previous electrocardiogram.
- Prescribed use of warfarin or direct-acting oral anticoagulant for thromboembolic stroke prevention.
- English-speaking at a a level appropriate for informed consent and study participation.
- Residence is defined as rural or in metropolitan Pittsburgh, Pennsylvania.
- No plans to relocate within 12 months of enrollment.

**Exclusion criteria**

- Conditions other than AF that require anticoagulation, such as mechanical prosthetic valve, deep vein thrombosis, or pulmonary embolism.
- Previous electrophysiologic treatment for AF, such as a pulmonary vein isolation.
- Heart failure necessitating hospital admission ≤3 months before study inclusion.
- Acute coronary syndrome (defined as at least 2 of the following: chest pain, ischemic electrocardiographic changes, or troponin≥0.1 ng/mL) ≤3 months before study inclusion.
- Untreated hyperthyroidism or ≤3 months euthyroidism before inclusion.
- Foreseen pacemaker, internal cardioverter defibrillator, or cardiac resynchronization therapy
- Cardiac surgery ≤3 months before inclusion.
- Planned cardiac surgery.
- Presence of noncardiovascular conditions likely to be fatal within 12 months (eg, cancer).
- Inability to comprehend the study protocol, defined by failing 3 times to correctly answer a set of questions during consent.
- A medical disorder, condition, or history that would impair the participant’s ability to participate or complete the study.

### Baseline Visit and Randomization

The study team obtained telephone-based informed consent at the start of the baseline visit. The informed consent outlined the study schedule, participant burden and compensation, access to the participant’s electronic health record, privacy and security protections in place for participant data in the electronic data management system, and provided contact information for the principal investigator of the study. In addition to providing telephone consent, participants returned a signed copy of the informed consent using a preaddressed, stamped envelope that accompanied study materials.

Consenting participants were randomized to intervention or control arms using electronic software [24,25]. Participants were provided with copies of assessments reproduced in 12- to 14-point font to enhance readability and facilitate their administration by telephone with trained assessors. All study materials were provided at a sixth grade reading level to ensure accessibility to participants. Table 1 lists the assessments conducted by study visit for both trials.

**Table 1.** Summary of assessments at trial time points (baseline 4, 8, and 12 months) in both randomized clinical trials.

Measure	Baseline	4 months	8 months	12 months
Demographics (age, sex, race, and ethnicity)	✓			
Primary physician for AF^a^ treatment	✓			
Transportation (car ownership, driver’s license, and distance to physician)	✓			
Mobile device proficiency and ownership [26]	✓			
Social and economic (annual household income, education, and marital status)	✓			
Social network and isolation [27]	✓			
Habits (tobacco and alcohol quantity)	**✓**			
Medications. Total number and schedules	✓			
AF history (duration and previous treatments)	✓			
Health literacy (Newest Vital Sign) [28,29]	✓			
Clinical conditions, comorbidities, and depression (PHQ-8)^c^ [30]				
PROMIS^d^ Self-efficacy [31]	✓	✓	✓	✓
Quality of life (AFEQT^e^ and PROMIS-29) [32,33]	✓	✓	✓	✓
Telephone Montreal Cognitive Assessment [34]	✓			✓
Medication adherence, self-report [35]	✓	✓	✓	✓
Proportion of days covered [36]		✓	✓	✓
Health care utilization		✓	✓	✓
New AF therapies and treatments^b^		✓	✓	✓
Qualitative interviews (relational agent, WebMD, and Kardia)		✓		

a AF therapies: pharmacologic or electrical cardioversion, electrophysiologic procedure such as pulmonary vein isolation, or initiation of antiarrhythmic medication.

b AF: atrial fibrillation.

c PHQ-8: Patient Health Questionnaire.

d PROMIS: Patient-reported Outcomes Measurement Information System.

e AFEQT: AF Effect on Quality of life.

### Smartphone Training and Intervention Content

Consented participants received study smartphones accompanied by training on their use and summary guides on smartphone and app operation developed for this study specific to the intervention and control arms. Materials provided to participants in the rural study and randomized to the intervention are provided as an Appendix in Multimedia Appendix 1. Training on smartphone and app use followed a standardized curriculum and concluded when participants reached capacity to operate the phone at a level appropriate for study participation. Smartphone ownership or digital literacy was consequently not required for trial participation. Smartphones were programmed to enable features such as phone calls and texting without the the capacity for more advanced functions such as downloading apps. Participants randomized to the intervention arms of the trials had the relational agent preinstalled on the smartphone and were provided detailed instructions regarding its use as part of smartphone instruction.

**Figure 1.**
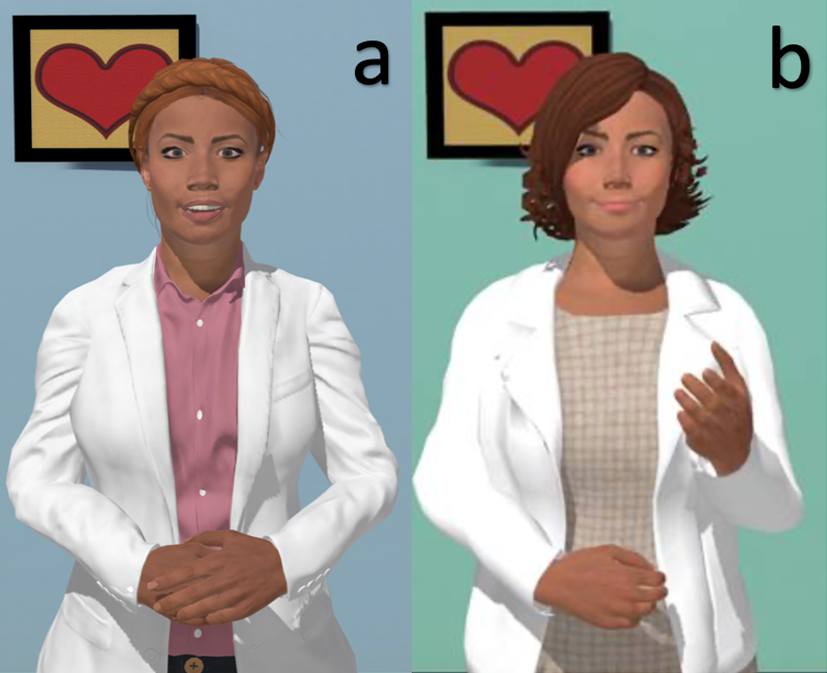
Visual representation of the relational agents that were used in the rural trial (Panel A) and metropolitan trial (Panel B) by intervention participants.

Relational agents are human-computer interventions adapted for multiple settings to facilitate education, problem-solving, and behavioral approaches with patients. Previous work has demonstrated that such agents are accessible to individuals with limited health and digital literacy with varied medical conditions [37-40]. Here, we designed the agent to provide education about AF; problem-solving regarding intentional and unintentional nonadherence to medications; preparation for the medical encounter; and address symptoms common in AF (eg, irregular or rapid heart rates, dyspnea, and chest discomfort). Relational agent content was modified for use in a rural or metropolitan setting. Figure 1 presents the 2 relational agents used in the trials. Intervention participants also received the AliveCor Kardia Mobile device [41] for heart rate and rhythm monitoring, as prompted by the relational agent, to reinforce self-care and the correlation of symptoms with heart rate and rhythm assessments.

Participants randomized to the trials’ control arms had the WebMD (WebMD LLC) app preinstalled on the study-provided smartphones. Research assistants encouraged participants to use this application to learn more about AF, its management, and the tracking of medications and symptoms. Participants in the control arms of both trials received an informational session from study team members providing a brief overview of AF and complications such as stroke, heart functioning, and signs of an impending stroke derived from American Heart Association educational materials. To further distinguish the 2 trials, control participants in the metropolitan trial also received the AliveCor Kardia Mobile device with instruction on its use, and guidance to use it as for heart rate and rhythm monitoring. In both studies, heart rate and rhythm obtained using this device was monitored, categorized, and recorded by the study team.

### Trial Outcomes

The trials shared the primary outcome to improve adherence to oral anticoagulation for thromboembolic stroke prevention in individuals with AF, as measured by pharmacy claims data using the proportion of days covered [36] (a validated measure to quantify medication adherence using pharmacy claims) and by participant self-report [35]. The secondary outcomes of the trials were (1) health-related quality of life [42], measured using the disease-specific AF Effect on Quality of life [33] and general patient-reported outcomes measurement information system [32] measures; and (2) health care utilization, as measured by days of hospitalization and emergency room visits.

### Participant Timeline and Data Collection

Both intervention and control applications were used for 4 months. Participants were sent a box with a prepaid label for returning the study phone and were informed that their second study payment was tied to a smartphone return. They were allowed to keep the Kardia Mobile device and received instruction from the study team on how to connect the device to their personal smartphone with the caveat that results would no longer be monitored by the study. Participants underwent repeat telephone assessments at 4-, 8-, and 12-months with simultaneous review of the electronic health record for hospitalization events. To assist with interviewer-administered phone-based instrument completion, participants were again mailed the packets of questionnaires summarized in Table 1.

### Remote Engagement and Retention

Given the absence of direct, personal contact, the study developed remote strategies for participant engagement. Participants received regular newsletters also written at 6th-grade reading level for the duration of the study that provided additional education about the studies and updates. In addition, the team mailed birthday cards to participants yearly throughout the duration of the study. Finally, study participants were offered the opportunity to participate in qualitative assessments to further share their experience of AF [43,44]. These sessions were conducted by experienced qualitative researchers using remote video conferencing software.

### Ethical Considerations

The trials described here were registered in clinicaltrials.gov with registration numbers NCT04076020 and NCT04075994 and were approved by the University of Pittsburgh institutional review board. All research participants provided informed consent that allows for secondary analyses without additional consent. This manuscript used solely deidentified data. Participants received compensation up to US $150 for participation across the 4 study visits.

## Results

**Figure 2.**
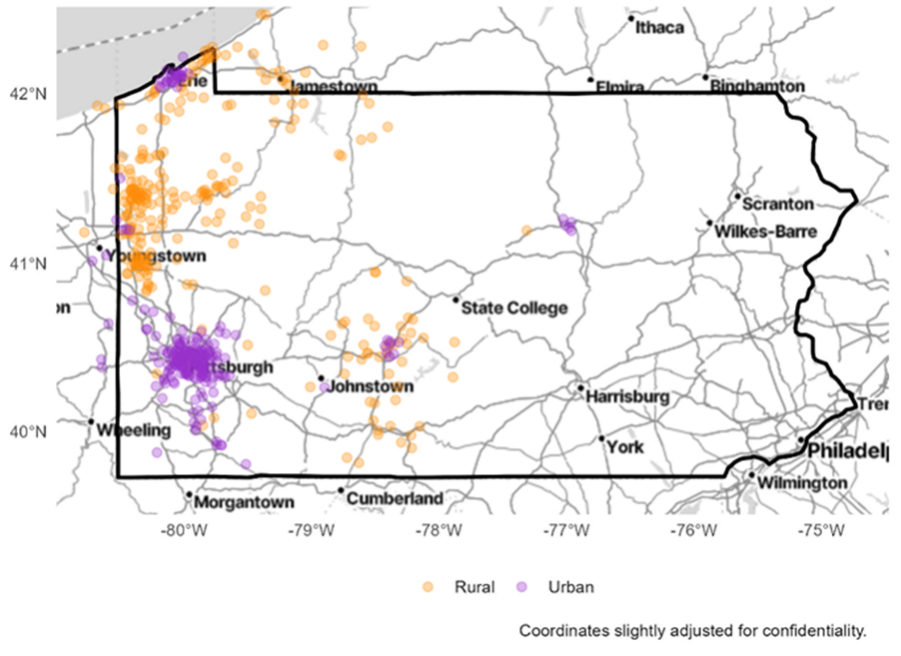
Map of the state of Pennsylvania with metropolitan resident participants in purple and rural participants in orange.

The rural study enrolled 270 participants while the metropolitan study enrolled 243 participants, in both instances surpassing enrollment goals. Figure 2 shows the geographic representation of participants according to their metropolitan or rural status.

Table 2 summarizes the demographic and social characteristics of participants in both studies. Each study enrolled >60% (319/513) women, consistent with the goal of enrolling individuals with limited participation in clinical trials for AF. In the rural study, 63.7% (172/270) of participants had an educational attainment level less than 4-year college, and 48.5% (131/270) had an annual household income of less than US $50,000/year. The metropolitan study included 30.5% (74/243) individuals of Black race, and 46.9% (114/243) of participants reported an educational attainment of less than 4-year college with 39.5% (96/243) having an annual household income less than US $50,000/year. In both studies, over 41% (225/513) of participants were categorized as having limited health literacy.

**Table 2.** Baseline characteristics of trial participants in rural Pennsylvania and the metropolitan Pittsburgh, Pennsylvania region.

Characteristic	Rural Pennsylvania (N=270)	Metropolitan Pittsburgh (N=243)
Age, years, median (min-max)	73.1 (40.8-92.2)	71.6 (29.7-89.6)
Years with AF^a^, mean (SD)	6.4 (7.7)	7.1 (8.1)
Sex		
Male	107 (39.6)	87 (35.8)
Female	163 (60.4)	156 (64.2)
Race		
White	257 (95.2)	161 (66.3)
Black	7 (2.6)	74 (30.5)
Asian	1 (0.4)	1 (0.4)
American Indian or Alaska Native	4 (1.5)	—
Multiple/Other	—	5 (2.1)
Unknown	4 (1.6)	2 (0.8)
Ethnicity		
Hispanic/Latino	5 (1.9)	0 (0)
Not Hispanic/Latino	261 (96.7)	240 (98.8)
Unknown	4 (1.4)	3 (1.2)
Education		
High School, vocational, or trade school	91 (33.7)	54 (22.2)
Vocational or trade School	37 (13.7)	18 (7.4)
Some college with no degree	44 (16.3)	42 (17.3)
Associate degree or higher	98 (36.3.)	117 (48.1)
Unknown	—	12 (4.9)
Employment status		
Employed, full or part-time	35 (13.0)	39 (16.0)
Retired	211 (78.2)	179 (73.7)
Other	24 (8.8)	25 (10.3)
Annual household income (US $)		
<19,999	33 (12.2)	29 (11.9)
20,000 to 34,999	52 (19.3)	35 (14.4)
35,000 to 49,999	46 (17.0)	32 (13.2)
50,000 to 74,999	42 (15.6)	39 (16.0)
75,000 to 99,999	27 (10.0)	27 (11.1 )
≥100,000	30 (11.1)	34 (14.0)
Do not know	40 (14.8)	45 (19.4)
Type of insurance		
Private	227 (84.1)	188 (77.4)
Public	41 (15.2)	54 (22.2)
None	2 (0.7)	1 (0.4)
Housing		
Ownership	185 (68.5)	137 (56.4)
Other status	85 (31.5)	106 (43.6)
Marital Status		
Married or living as married	187 (69.2)	121 (49.8)
Widowed	49 (18.2)	45 (18.5)
Separated or divorced	34 (12.6)	77 (31.7)
AF, selection of anticoagulant medication		
Warfarin	46 (17.0)	34 (14.0)
Direct oral anticoagulant	225 (83.3)	209 (86)
Health literacy		
Limited health literacy	125 (46.3)	100 (41.2)
Adequate health literacy	145 (53.7)	143 (58.8)

a AF: atrial fibrillation.

Participants randomized to the intervention demonstrated excellent fidelity regarding the use of the relational agent. Rural individuals employed the agent for a median of 101 (IQR, 72‐110) days of the 120-day trial. Likewise, those randomized to the intervention arm of the metropolitan cohort used the agent for a median of 98 (IQR 58‐109) days of the 120-day trial. Median days of AliveCor Kardia Mobile device use was 102 (IQR 109‐123) in the rural trial relative to 95 (IQR 62‐109) in the metropolitan trial. Out of 270, 239 (88.5%) and 218 (89.7%) participants completed 12-month assessments in the rural and metropolitan trials, respectively.

**Figure 3.**
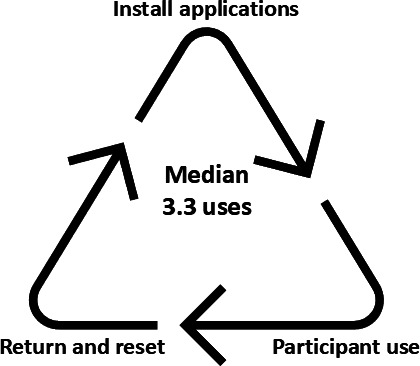
Progression of smartphone distribution in the clinical trials.

Phones were returned at 4 months, cleaned, and then reused for additional participants. Figure 3 graphically summarizes the distribution of smartphones. Between the 513 participants of the 2 trials, there were a total of 165 smartphones used with a median use of 3.3 (range 1‐5) trial participants. In total, 16 phones were lost, stolen, or broken, 5 of which were lost by mail delivery services, not the participant. In addition, 3 phones were lost to participants who withdrew or died without returning the phone to the study.

## Discussion

### Principal Findings

The decentralized research program described here demonstrates the successful enrollment of rural and metropolitan individuals with AF in clinical trials using a mobile health intervention. Our approach for remote engagement yielded geographic and racial diversity in study participants that exceeds many AF trials. Both trials fulfilled enrollment goals with participants maintaining excellent fidelity to the relational agent intervention and to the AliveCor Kardia Mobile device. Our studies consequently affirm the feasibility of conducting remote, decentralized trials with mobile health interventions, and affirm the demonstrated capacity of decentralized trials to enhance participant diversity [45,46].

### AF and Mobile Health: In the Context of the Literature

AF is a complex syndrome treated with long-term, possibly lifelong, oral anticoagulation for stroke prevention, and managed with subspecialty care and procedures according to professional society guidelines [42]. Our program aimed to address the prominent challenges of health literacy, medication adherence, quality of life, and increased health care utilization that individuals with AF may experience. Our program was informed further by the consistent literature demonstrating the prominent associations of social and structural factors with care processes in AF and its related outcomes [47,48]. Community-based studies, registries, and health services analyses have identified that individuals of non-White race, lower educational attainment, lower income, and residence in neighborhoods with greater social deprivation experience more clinical adversity and limited access to AF-related care than their counterparts [15,16,49-52]. However, social and structural factors are not regularly captured in the conduct of research related to AF, potentially perpetuating disparities by precluding assessment of generalizability to individuals and populations that experience greater social disadvantage. By enrolling individuals with limited education and social resources, our program sought to enhance the access and generalizability of our research program.

Our approach further eliminated geographic and financial barriers to participation. As the intervention was delivered via smartphone, we provided smartphones to participants for the study period along with standardized instruction for their use, thereby eliminating access to contemporary technology as a barrier to trial participation. Our provision of study materials at a sixth-grade reading level and verbal administration of surveys by staff further diminished health literacy as an obstacle to engagement. Telephone-based visits likewise reduced geographic distance and travel as obstacles to participation. A further iteration of our programmatic design may include video-based visits and additional phenotypic characterization of study participants. Finally, as the literature documents both the increased adversity in women with AF accompanied by diminished participation in clinical trials [53-55], we emphasized the recruitment of women, achieving >60% (319/513) enrollment of women in both trials.

### Promise and Pitfalls of Trials Using Digital Interventions

Remote trials have promise to implement novel digital interventions, such as the relational agent used here. Advantages include the provision of patient-centered education, relevant monitoring, availability, and increased attention and feedback to promote self-care. A meta-analysis determined that digital interventions have the potential to increase medication adherence–the primary outcome of our studies–by 10% (95% CI 1.00‐1.22) [56].

Concerns for the implementation of digital trials include technological literacy, access to services, and sustainability. Implementation of digital interventions necessitates attention to digital literacy, addressed here by the provision of standardized education and staff support regarding smartphone and device use. Increased dependence on technologies has necessitated the use of digital devices for communication and health maintenance but challenges approximately 20% of individuals reported limited digital literacy in one convenience sample [57]. In addition, the provision of digital technologies requires infrastructure for their effective use. Persistent disparities in Broadband access and coverage present an additional obstacle to the effective implementation of trials using digital technologies. Our study, conducted in metropolitan and rural Pennsylvania, benefited from most participants having adequate cellular coverage and access to the relational agent not being dependent on connectivity. Despite providing access to smartphones, several participants experienced challenges during the trial such as software updates and complications during use as is typical for mobile health trials.

The provision of smartphones to participants eliminated technology access as a barrier to participation. However, we recognize that such an approach would be challenging to sustain beyond the duration of the clinical trials described here. More sustained deployment of mobile health intervention requires assessment of (1) a budget impact, to appreciate the long-term costs (including technology infrastructure and maintenance) and savings of such an intervention and (2) further assessment of the facilitators and barriers that inform the implementation process of the intervention. The next steps of our program include evaluating the implementation process and ascertaining its cost-effectiveness.

### Strengths and Limitations

We recognize several strengths of our programs. We conducted 2 decentralized trials that used a digital health intervention, exceeding recruitment goals in rural and metropolitan settings. Intervention participants demonstrated excellent fidelity with use of the relational agent. Our program also has important, noteworthy limitations that we consider foremost as pertinent to generalizability. First, we recognize rurality as highly heterogeneous and expect that our cohort of rural individuals is not representative of those in other rural contexts. Second, the rural trial was primarily White race, reflecting the region’s demographic composition, but again limiting the generalizability of our findings to more racially and ethnically diverse populations. In contrast, the metropolitan study recruited 30% (74/243) individuals of Black race and hence demonstrated greater racial diversity. Third, other settings may benefit from relational agents that are tailored for regional factors such as culture, traditions, digital services, and social and structural factors. We recognize the expansion of agent content as a priority for its implementation in other settings. Together, location, demographic composition, and relational agent design contribute to the limited generalizability of our trials. Finally, the remote design and conduct of assessments by telephone, albeit eliminating multiple obstacles, may be accompanied by a decreased opportunity for more extensive participant characterization and assessments. Remote trial investigators must balance the potential to eliminate participation barriers with the capacity to obtain more robust participant phenotyping and measurements.

### Conclusions

We developed a decentralized, remote research program using a digital intervention. We successfully recruited and enrolled diverse participants that contrast with the relative geographic and social homogeneity of many clinical trials for AF. We intend for our program to provide a roadmap for attaining diverse study participation in digital interventions in decentralized clinical trials for chronic cardiovascular and noncardiovascular diseases.

## Supplementary material

10.2196/66436 Multimedia Appendix 1 Summary of instructions for smartphone and app use accompanied by standardized instruction.

## Abbreviations

AF: atrial fibrillation

## References

[1] Michos ED, Reddy TK, Gulati M (2021). Improving the enrollment of women and racially/ethnically diverse populations in cardiovascular clinical trials: An ASPC practice statement. Am J Prev Cardiol.

[2] Tahhan AS, Vaduganathan M, Greene SJ (2020). Enrollment of older patients, women, and racial/ethnic minority groups in contemporary acute coronary syndrome clinical trials: a systematic review. JAMA Cardiol.

[3] Khan MS, Shahid I, Siddiqi TJ (2020). Ten-year trends in enrollment of women and minorities in pivotal trials supporting recent US food and drug administration approval of novel cardiometabolic drugs. J Am Heart Assoc.

[4] Breathett K, Sims M, Gross M (2020). Cardiovascular health in American Indians and Alaska Natives: a scientific statement from the american heart association. Circulation.

[5] Liu KA, Mager NAD (2016). Women’s involvement in clinical trials: historical perspective and future implications. Pharm Pract (Granada).

[6] Khan SU, Khan MZ, Raghu Subramanian C (2020). Participation of women and older participants in randomized clinical trials of lipid-lowering therapies: a systematic review. JAMA Netw Open.

[7] Oh SS, Galanter J, Thakur N (2015). Diversity in clinical and biomedical research: a promise yet to be fulfilled. PLoS Med.

[8] Aggarwal NR, Patel HN, Mehta LS (2018). Sex differences in ischemic heart disease: advances, obstacles, and next steps. Circ Cardiovasc Qual Outcomes.

[9] Tertulien T, Magnani JW, Essien UR (2021). Racial and ethnic representation in atrial fibrillation trials: CABANA and beyond. J Am Coll Cardiol.

[10] Zimetbaum P (2017). Atrial fibrillation. Ann Intern Med.

[11] Kornej J, Börschel CS, Benjamin EJ, Schnabel RB (2020). Epidemiology of atrial fibrillation in the 21st century: novel methods and new insights. Circ Res.

[12] Jiang S, Seslar SP, Sloan LA, Hansen RN (2022). Health care resource utilization and costs associated with atrial fibrillation and rural-urban disparities. J Manag Care Spec Pharm.

[13] Deshmukh A, Iglesias M, Khanna R, Beaulieu T (2022). Healthcare utilization and costs associated with a diagnosis of incident atrial fibrillation. Heart Rhythm O2.

[14] Kim MH, Johnston SS, Chu BC, Dalal MR, Schulman KL (2011). Estimation of total incremental health care costs in patients with atrial fibrillation in the United States. Circ Cardiovasc Qual Outcomes.

[15] Essien UR, Kornej J, Johnson AE, Schulson LB, Benjamin EJ, Magnani JW (2021). Social determinants of atrial fibrillation. Nat Rev Cardiol.

[16] Essien UR, Kim N, Hausmann LRM (2021). Disparities in anticoagulant therapy initiation for incident atrial fibrillation by Race/Ethnicity among patients in the Veterans Health Administration System. JAMA Netw Open.

[17] Riegel B, Moser DK, Buck HG (2017). Self-care for the prevention and management of cardiovascular disease and stroke: a scientific statement for healthcare professionals from the American Heart Association. J Am Heart Assoc.

[18] Althouse AD, Abebe KZ, Paasche-Orlow MK (2023). Design, rationale, and baseline characteristics of a randomized controlled trial evaluating a mobile relational agent to enhance atrial fibrillation self-care. Contemp Clin Trials.

[19] Dorsey ER, Kluger B, Lipset CH (2020). The new normal in clinical trials: decentralized studies. Ann Neurol.

[20] Schwartz AL, Alsan M, Morris AA, Halpern SD (2023). Why diverse clinical trial participation matters. N Engl J Med.

[21] Bickmore T, Schulman D, Yin L (2010). Maintaining engagement in long-term interventions with relational agents. Appl Artif Intell.

[22] Magnani JW, Ferry D, Swabe G (2022). Design and rationale of the mobile health intervention for rural atrial fibrillation. Am Heart J.

[23] Magnani JW, Ferry D, Swabe G (2021). Rurality and atrial fibrillation: a pathway to virtual engagement and clinical trial recruitment in response to COVID-19. Am Heart J Plus.

[24] Harris PA, Taylor R, Minor BL (2019). The REDCap consortium: Building an international community of software platform partners. J Biomed Inform.

[25] blockrand SG Randomization for block random clinical trials.

[26] Roque NA, Boot WR (2018). A new tool for assessing mobile device proficiency in older adults: the mobile device proficiency questionnaire. J Appl Gerontol.

[27] Berkman LF, Syme SL (1979). Social networks, host resistance, and mortality: a nine-year follow-up study of Alameda County residents. Am J Epidemiol.

[28] Chew LD, Bradley KA, Boyko EJ (2004). Brief questions to identify patients with inadequate health literacy. Fam Med.

[29] Weiss BD, Mays MZ, Martz W (2005). Quick assessment of literacy in primary care: the newest vital sign. Ann Fam Med.

[30] Kroenke K, Strine TW, Spitzer RL, Williams JBW, Berry JT, Mokdad AH (2009). The PHQ-8 as a measure of current depression in the general population. J Affect Disord.

[31] Lorig KR, Sobel DS, Ritter PL, Laurent D, Hobbs M (2001). Effect of a self-management program on patients with chronic disease. Eff Clin Pract.

[32] Cella D, Riley W, Stone A (2010). The patient reported outcomes measurement information system (PROMIS) developed and tested its first wave of adult self-reported health outcome item banks: 2005-2008. J Clin Epidemiol.

[33] Spertus J, Dorian P, Bubien R (2011). Development and validation of the atrial fibrillation effect on quality-of-life (AFEQT) Questionnaire in patients with atrial fibrillation. Circ Arrhythm Electrophysiol.

[34] Katz MJ, Wang C, Nester CO (2021). T-MoCA: A valid phone screen for cognitive impairment in diverse community samples. Alzheimers Dement (Amst).

[35] Voils CI, Maciejewski ML, Hoyle RH (2012). Initial validation of a self-report measure of the extent of and reasons for medication nonadherence. Med Care.

[36] Peterson AM, Nau DP, Cramer JA, Benner J, Gwadry-Sridhar F, Nichol M (2007). A checklist for medication compliance and persistence studies using retrospective databases. Value Health.

[37] Bickmore TW, Silliman RA, Nelson K (2013). A randomized controlled trial of an automated exercise coach for older adults. J Am Geriatr Soc.

[38] Bickmore TW, Utami D, Matsuyama R, Paasche-Orlow MK (2016). Improving access to online health information with conversational agents: a randomized controlled experiment. J Med Internet Res.

[39] King AC, Bickmore TW, Campero MI, Pruitt LA, Yin JL (2013). Employing virtual advisors in preventive care for underserved communities: results from the COMPASS study. J Health Commun.

[40] King AC, Campero MI, Sheats JL (2020). Effects of counseling by peer human advisors vs computers to increase walking in underserved populations: The COMPASS randomized clinical trial. JAMA Intern Med.

[41] Wegner FK, Kochhäuser S, Ellermann C (2020). Prospective blinded evaluation of the smartphone-based AliveCor Kardia ECG monitor for atrial fibrillation detection: The PEAK-AF study. Eur J Intern Med.

[42] Joglar JA, Chung MK, Armbruster AL, Benjamin EJ, Chyou JY, Writing Committee Members (2024). 2023 ACC/AHA/ACCP/HRS Guideline for the diagnosis and management of atrial fibrillation: a report of the American college of cardiology/American Heart Association Joint Committee on clinical practice guidelines. J Am Coll Cardiol.

[43] Mann H, Johnson AE, Ferry D (2023). A qualitative crossroads of rhythm and race: Black patients’ experiences living with atrial fibrillation. Am Heart J Plus.

[44] Mann HK, Streiff M, Schultz KC (2023). Rurality and atrial fibrillation: patient perceptions of barriers and facilitators to care. J Am Heart Assoc.

[45] Mayfield JJ, Chatterjee NA, Noseworthy PA (2021). Implementation of a fully remote randomized clinical trial with cardiac monitoring. Commun Med (Lond).

[46] Stewart J, Krows ML, Schaafsma TT (2022). Comparison of racial, ethnic, and geographic location diversity of participants enrolled in clinic-based vs 2 remote COVID-19 clinical trials. JAMA Netw Open.

[47] Martin SS, Aday AW, Almarzooq ZI (2024). 2024 heart disease and stroke statistics: a report of US and global data from the American Heart Association. Circulation.

[48] Benjamin EJ, Thomas KL, Go AS (2023). Transforming atrial fibrillation research to integrate social determinants of health: a National Heart, Lung, and Blood Institute workshop report. JAMA Cardiol.

[49] Essien UR, Chiswell K, Kaltenbach LA (2022). Association of race and ethnicity with oral anticoagulation and associated outcomes in patients with atrial fibrillation: findings from the get with the guidelines-atrial fibrillation registry. JAMA Cardiol.

[50] Magnani JW, Norby FL, Agarwal SK (2016). Racial differences in atrial fibrillation-related cardiovascular disease and mortality: the atherosclerosis risk in communities (ARIC) study. JAMA Cardiol.

[51] Tertulien T, Chen Y, Althouse AD, Essien UR, Johnson AE, Magnani JW (2021). Association of income and educational attainment in hospitalization events in atrial fibrillation. Am J Prev Cardiol.

[52] Abdel-Qadir H, Akioyamen LE, Fang J (2022). Association of neighborhood-level material deprivation with atrial fibrillation care in a single-payer health care system: a population-based cohort study. Circulation.

[53] Emdin CA, Wong CX, Hsiao AJ (2016). Atrial fibrillation as risk factor for cardiovascular disease and death in women compared with men: systematic review and meta-analysis of cohort studies. BMJ.

[54] Piccini JP, Simon DN, Steinberg BA (2016). Differences in clinical and functional outcomes of atrial fibrillation in women and men: two-year results from the ORBIT-AF registry. JAMA Cardiol.

[55] Khan SU, Raghu Subramanian C, Khan MZ (2022). Association of women authors with women enrollment in clinical trials of atrial fibrillation. J Am Heart Assoc.

[56] Akinosun AS, Polson R, Diaz-Skeete Y (2021). Digital technology interventions for risk factor modification in patients with cardiovascular disease: systematic review and meta-analysis. JMIR Mhealth Uhealth.

[57] Jongebloed H, Anderson K, Winter N (2024). The digital divide in rural and regional communities: a survey on the use of digital health technology and implications for supporting technology use. BMC Res Notes.

