# Supplementary material for: Augmenting Engagement in Decentralized Clinical Trials for Atrial Fibrillation: Development and Implementation of a Programmatic Architecture
Source: JMIR Cardio. 2025 May 12;9:e66436. doi: 10.2196/66436 (PMC12088620; doi:10.2196/66436)

AFib LITT

Using your Study iPhone

A Step by Step Guide

What’s Inside

Use this Guide To:

Use the iPhone

Turn the iPhone On...1
Turn the iPhone Off...1
Charge the iPhone...2
Change the Volume...2
Return to the Main Screen...3
Make a phone call..3
Answer the phone when it rings..................................4
Find the phone number for this iPhone.....................4

To Turn the iPhone On:

1. Press and hold in the button on the top-right side of the iPhone until the Apple logo appears. *Note: This will take several seconds.*
2. Release the button when the Apple logo appears. Your home screen will appear next.

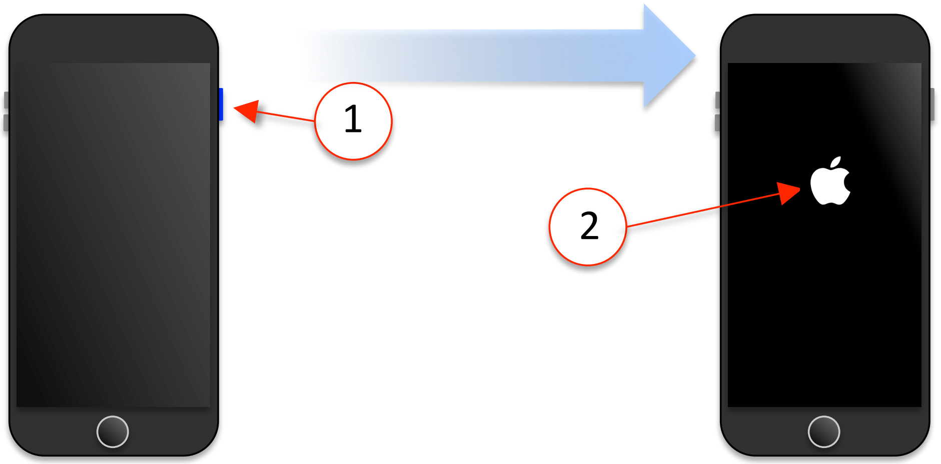

To Turn the iPhone Off:

1. Press and hold in the button on the top-right side of the iPhone. This will take several seconds.
2. Release the button when a red circle appears followed by a message that says “slide to power off.”
3. Touch the red circle and slide it with your finger to the edge of the phone. If you find this hard to do, pretend you are moving a bread crumb across the screen and off of the phone. It’s the same movement.

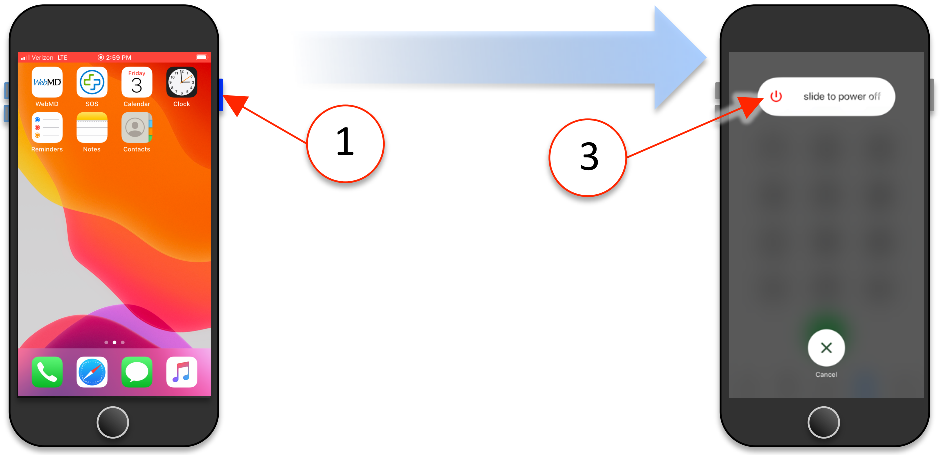

To Charge the iPhone:

1. Using the charger that came with the phone, firmly push the small metal end into slot at the bottom of the iPhone.

2. Plug the other end into a wall outlet. If it is easier for you, you can leave the phone plugged into the charger so that you don't misplace it and it is ready to charge whenever you need to.

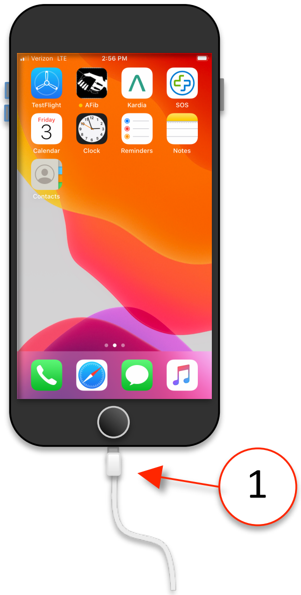

To Change the Volume:

1. To raise the volume, press the small top button on the left side of the iPhone until the desired volume is reached.

2. To lower the volume, press the small bottom on the left side of the iPhone until the desired volume is reached.

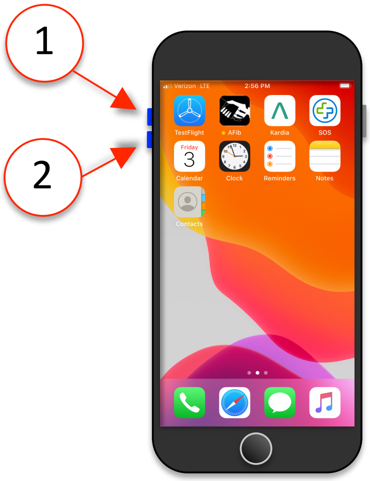

To Return to the Main Screen:

If the screen of the iPhone goes black, or if you want to quit whatever you're doing on the phone and get back to a safe, familiar screen, you can easily do that by pressing the "Home" button.

1. Locate the round button at the bottom of the phone and give it one, firm, quick push.
2. Your "home" screen will appear. Your home screen is where you will find the buttons, or "apps," that start all of the activities that you do most often.

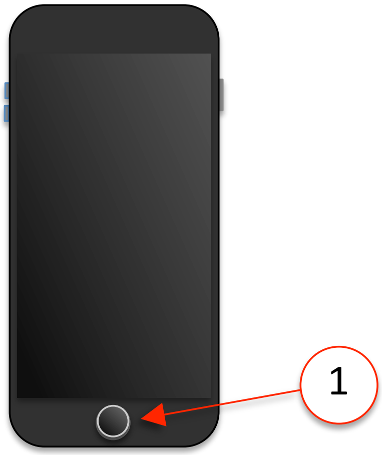

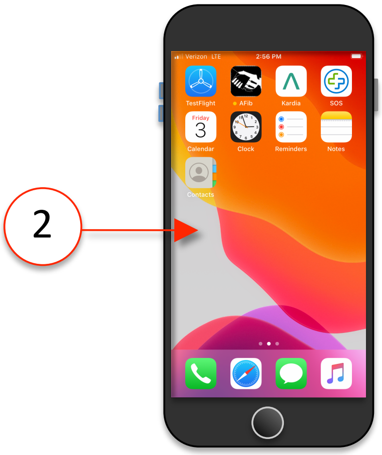

To Make a Phone Call:

1. Tap the green phone button with your finger one time – just a light tap. The Phone app will appear.
2. Tap the Keypad button on the bottom of the screen. A numbered keypad will appear.
3. Tap the number into the keypad and then tap the green phone button to send the call.
4. When the call is made, the phone button turns red. Tap it to end the call at any time.

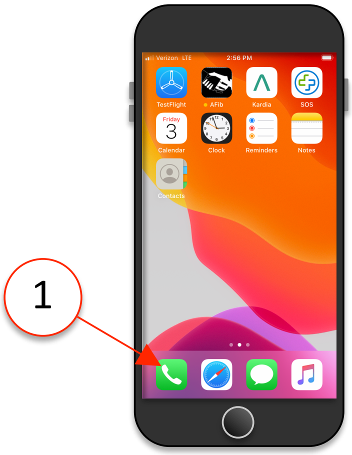

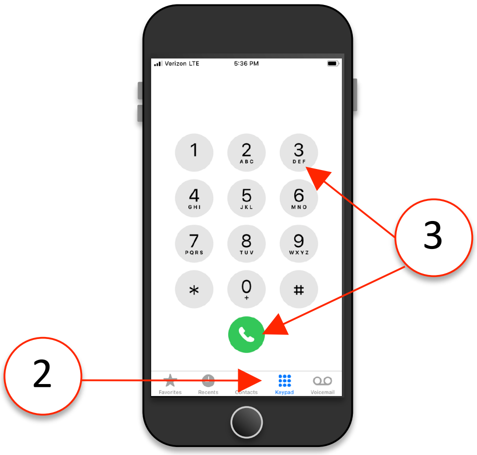

To Answer a Phone Call:

When your phone rings you can choose to answer it or to decline it.

1. To answer the call, tap the green answer button.
2. To decline the call, tap the red

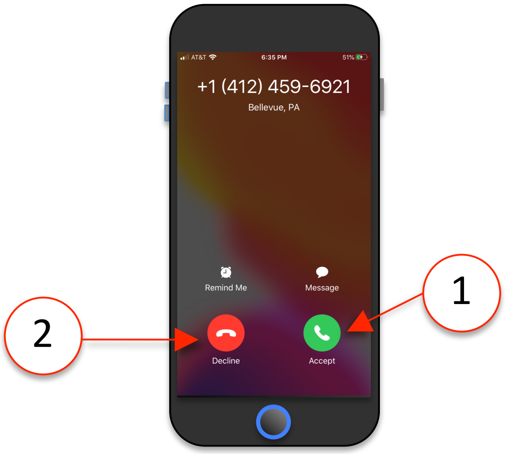

To Find the Number for This Phone:

1. Tap the green phone button with your finger one time – just a light tap. The Phone app will appear.
2. Tap the Contact button on the bottom of the screen. A list of your contacts will appear.
3. Tap on the AFib Lit contact. Your contact card will appear.
4. Locate the phone number and email address you can be contacted by on this phone.

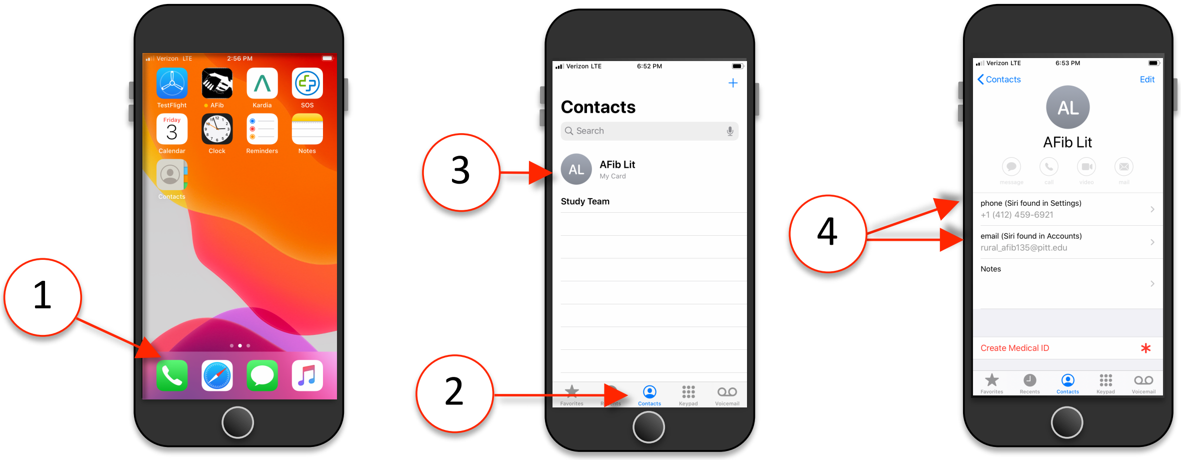

Supplement: Multimedia Appendix 1 [file cardio-v9-e66436-s001.pdf]
